# The Soluble Platelet‐Derived Growth Factor β Receptor Induces Postoperative Delirium by Downregulating the Clearance of β‐Amyloid in the Brain

**DOI:** 10.1002/brb3.70436

**Published:** 2025-05-05

**Authors:** Zongfeng Guo, Chen Zhang, Xiang Wang, Weiguo Chen, Zongxiao Guo

**Affiliations:** ^1^ Anesthesiology Department Hai 'an People's Hospital Affiliated to Nantong University Hai'an China; ^2^ Central Laboratory Hai 'an People's Hospital Affiliated to Nantong University Hai'an China; ^3^ Department of Orthopaedics Surgery Hai 'an People's Hospital Affiliated to Nantong University Hai'an China

**Keywords:** blood–brain barrier, cognitive function, postoperative delirium, sPDGFRβ

## Abstract

**Purpose:**

To investigate the relationship between soluble platelet‐derived growth factor β receptor (sPDGFRβ) in cerebrospinal fluid (CSF) and Alzheimer's disease (AD) biomarkers, to determine whether high CSF sPDGGFRβ is a potential risk factor for postoperative delirium (POD), and to evaluate its predictive effect, so as to provide reference for clinical prevention and treatment.

**Patients and Methods:**

CSF samples were collected preoperatively from cognitively normal participants aged 50–90 years undergoing knee/hip replacement surgery under spinal‐epidural anesthesia. The concentrations of sPDGFRβ, β‐amyloid 42 (Aβ42), total tau protein (Ttau), and phosphorylated tau protein (Ptau) in CSF were detected by enzyme‐linked immunosorbent assay (ELISA). The confusion assessment method (CAM) was used to evaluate whether participants developed POD after surgery, and they were divided into the POD group and non‐POD group (NPOD). The relationship between CSF sPDGFRβ, AD biomarkers, and POD was analyzed.

**Results:**

The level of sPDGFRβ, a marker of brain pericyte injury, was significantly increased in POD patients (*p* < 0.05), and the difference was still statistically significant after adjusting for multiple confounders (*p* < 0.05). CSF Aβ42 showed a significant mediating effect between CSF sPDGFRβ level and POD (22.45%). The combination of AD biomarkers and CSF sPDGFRβ predicted POD better than that of AD biomarkers or CSF sPDGFRβ alone.

**Conclusion:**

The results suggest that the increase in CSF sPDGFRβ is associated with an increased risk of POD due to the blood–brain barrier (BBB) dysfunction and reduced Aβ42 clearance. In this study, the correlation between CSF sPDGFRβ and POD was investigated for the first time, providing a new reference index for POD prediction. However, this paper did not study other relevant indicators of the BBB and lacked follow‐up, which could be further improved in the future.

## Graphical abstract

Brain pericyte injury may contribute to the onset and progression of POD by influencing the blood–brain barrier's capacity to clear Aβ protein. The combination of CSF sPDGFRβ with CSF AD biomarkers exhibits improved predictive abilities for POD.

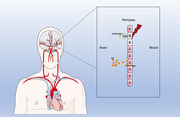

## 1 Introduction

Postoperative delirium (POD) emerges as a frequent neurological hassle in elderly patients, representing a nonspecific parenchymal brain syndrome (Swarbrick and Partridge 2022). POD is an acute, reversible mental disorder that involves sudden shifts in mental state, attention disorders, confusion, varying levels of consciousness disorders, loss of orientation, mood swings, and sleep‐wake disorders. Although most cases of POD are transient and self‐limited, the condition can yield many adverse effects on patient outcomes (Oh et al. 2017). At present, the primary focus remains on preventing POD, emphasizing the critical need to investigate the contributing factors for this postoperative cognitive impairment (Duning et al. 2021).

Cerebrospinal fluid (CSF), indicative of Alzheimer's disease (AD) biomarkers such as β‐amyloid 40 (Aβ40), β‐amyloid 42 (Aβ42), phosphorylated tau (Ptau), and total tau (Ttau), is closely related to AD, POD, and other central nervous system diseases (Barthélemy et al. 2020; Reiss et al. 2018; Salvadores et al. 2020; Yan et al. 2020). Aβ is a substantial protein molecule originating from the lipid membrane of nerve cells. Upon reaching a specific concentration, it undergoes aggregation, leading to the formation of small protein clusters that progressively accumulate and give rise to plaques (Wang et al. 2017). Tau protein serves as a microtubule‐affiliated protein, with a principal role in upholding the stability of axonal microtubules and guaranteeing the regular functioning of brain activities. In instances where tau undergoes hyperphosphorylation, its capacity to adhere to microtubules diminishes, culminating in the development of neurofibrillary tangles (Kandimalla et al. 2018). In this study, we suggest that Aβ plaque deposition and tau protein fiber entanglement could lead to nervous system failure and cognitive decline (Quiroz et al. 2018).

The blood–brain barrier (BBB) comprises cells of the vascular endothelial and supporting kinds, serving to regulate the exchange of blood–brain substances and protect the central nervous system from neurotoxins and pathogens (Abbott et al. 2010). Located within the capillary walls of the brain, brain pericytes have a crucial part in maintaining the structural and functional integrity of the BBB and protecting neuronal and cognitive function (Armulik et al. 2010; Hirunpattarasilp et al. 2019). Recent studies have found that pericytes, when separated from microvessels, can cause inflammatory immune responses, leading to BBB damage and brain edema (Rustenhoven et al. 2017). On the other hand, alterations in the level of soluble platelet‐derived growth factor β receptor (sPDGFRβ) in CSF have been linked to brain pericyte injury, including a reduction in their number, thus serving as a marker for pericyte‐related damage (Montagne et al. 2015; Nation et al. 2019). Current studies have shown that damage to brain pericytes is associated with the advancement of individuals undergoing cognitive impairment and AD (Sweeney et al. 2020; Wang et al. 2022). However, the connection between CSF sPDGFRβ, AD biomarkers, and its role in POD is yet to be elucidated.

Here, our objective was to explore the association between CSF sPDGFRβ levels and the BBB capability to clear Aβ and Tau, to ultimately ascertain whether elevated CSF sPDGFRβ serves as a potential risk factor for POD.

## 2 Material and Methods

### 2.1 Participants

This study collected information on individuals having knee/hip replacement surgery with spinal‐epidural anesthesia at Hai ‘an People's Hospital, affiliated to Nantong University, between October 2022 and May 2023. Ethical approval was obtained from the Ethics Committee of Hai ’an People's Hospital, affiliated to Nantong University (Clinical registration number: ChiCTR2200064740). Written informed consent was acquired from patients or the corresponding legal representatives for the collection of CSF samples. The study adheres to the principles outlined in the Declaration of Helsinki.

Inclusion criteria for participant selection encompassed the following: (1) Age between 50 and 90 years; (2) American Society of Anesthesiologists (ASA) physical status classification I–III; (3) Undergoing combined epidural anesthesia. Exclusion criteria encompassed: (1) Mini‐Mental State Examination (MMSE) scores below 24 preoperatively; (2) Presence of cancer; (3) Severe heart, liver, or renal insufficiency; (4) Neurological disorders and mental illnesses (e.g., stroke, Parkinson's syndrome, schizophrenia); (5) Unwillingness to engage in the study.

**FIGURE 1:**
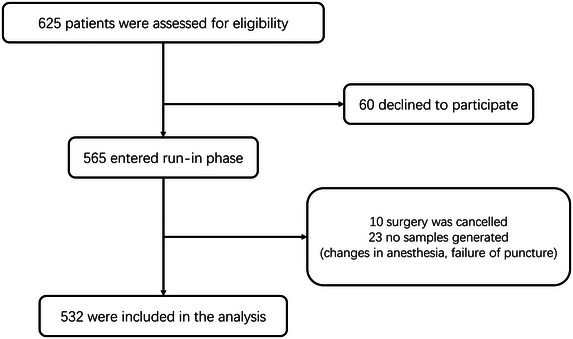
Participants’ recruitment chart.

Demographic details such as gender, age, years of education, MMSE scores, diabetes, hypertension, alcohol consumption, and smoking were documented before the surgical procedure. The preliminary examination identified 10 covariates expected to be included in the logistic regression. Assuming a 20% incidence of POD and accounting for an estimated 20% loss to follow‐up, the estimated sample size needed was 625 cases [10 × 10 ÷ 0.2 ÷ (1 − 0.2)] (van Smeden et al. 2019). Following the screening process, a total of 532 individuals were ultimately enrolled in this study (Figure 1).

### 2.2 CSF Sampling and Processing

The patients refrained from short‐term drug treatment and adhered to the medical recommendation to abstain from alcohol and fast 1 day before the surgery. Upon entering the operating room, standard monitoring of vital signs such as pulse oxygen saturation (SpO2), electrocardiogram (ECG), and noninvasive blood pressure (NBP) was initiated. Venous access was established, and a continuous infusion of a compound sodium chloride solution was administered at a constant rate.

The anesthesia position adopted was lateral decubitus, and a single‐segment technique (SST) was employed for puncture, specifically targeting the L3–L4 region. Following a successful puncture, 5 mL of CSF was extracted from the subarachnoid space. Subsequently, 2–2.5 mL (0.66%) of ropivacaine was injected, an epidural catheter was inserted, and an additional 3–5 mL of 2% lidocaine was administered as needed throughout the operation. Postanesthesia, the sensory level was meticulously monitored at the T8‐S5 level. Throughout the procedure, oxygen was administered via a mask, and vasoactive drugs were judiciously administered based on fluctuations in blood pressure and heart rate, ensuring the maintenance of stable vital signs.

Following completion of the operation, participants were transferred to the postanesthesia care unit (PACU) for a 30 min observation period. If no abnormalities were detected, patients were then returned to the ward. CSF was processed in accordance with the guidelines of the Alzheimer's Biomarker Standardization Program (ABSI) (Vanderstichele et al. 2012). CSF samples were centrifuged (2000 × *g*, 10 min) within 2 h after collection. The resultant CSF supernatant was then preserved in an enzyme‐free EP (Eppendorf) tube (AXYGEN; PCR‐02‐C) at −80°C for subsequent analysis.

### 2.3 CSF Measurement

Strictly follow the instructions. CSF Aβ42, Ttau, and Ptau levels were assessed employing an enzyme‐linked immunosorbent assay (ELISA) kit (INNOTEST, Fujirebio, Belgium). CSF sPDGFRβ levels were determined using a human sPDGFRβ ELISA kit (Thermo Scientific, Massachusetts, USA). Every sample within this study underwent analysis by proficient technicians utilizing identical methodologies. The measurement outcomes were obtained in triplicate and subsequently averaged for statistical analysis.

### 2.4 Cognitive Assessment

Cognitive function assessment for patients occurred 1 day before surgery using the MMSE. Individuals with MMSE scores below 24 were removed from the study. The occurrence of POD was assessed using the Confusion Assessment Method (CAM) two times a day at 8:00–9:00 a.m. and 3:00–4:00 p.m. at 6 h, 1 day, 3 days, and 7 days postsurgery (Breitbart et al. 1997; Inouye et al. 1990). Patients were screened until they had POD or the follow‐up period ended. CAM, recognized as the most frequently used and effective technique for delirium screening, is based on four clinical criteria for POD diagnosis: (1) acute onset and fluctuating course; (2) inattention; (3) disorganized thinking; (4) altered level of consciousness. Delirium is defined when both (1) and (2) are met concurrently, along with either (3) and/or (4). This diagnostic method offers a straightforward, rapid, and accurate clinical assessment for nonpsychiatric clinicians, demonstrating high sensitivity (94%–100%) and specificity (90%–95%) (Wei et al. 2008). When patients were assessed for POD, the severity of the pod was assessed using the Memorial Delirium Assessment Scale (MDAS). All neuropsychological tests in this study were administered by clinicians with relevant learning and training.

### 2.5 Statistics Analyses

Statistical analysis utilized the IBM SPSS 22.0 software for Windows (SPSS; USA), R‐4.1.0 (R Foundation for Statistical Computing, Austria), employing packages such as ggplot2, ggpubr, RColorBrewer, and mediation. Additionally, MedCalc (v22.009) was employed for specific analyses. To assess the normal distribution of demographic data, the Kolmogorov–Smirnov test was applied. Measurement data with a normal distribution were presented as mean ± standard deviation (x ± s), and the comparison between groups employed the *t*‐test for two independent samples. For skewed distribution measurement data, the median (interquartile range) [M (Q)] was used, and the Mann–Whitney *U* test was applied for group comparisons. Chi‐square tests were employed for comparisons involving categorical data.

Participants were categorized into two groups: the POD group and the non‐POD group (NPOD), based on the occurrence of POD after the operation. Differences in CSF sPDGFRβ, Aβ42, Ttau, and Ptau levels between these groups were analyzed. To mitigate the impact of confounding factors, univariate binary logistic regression was employed to examine the relationship between CSF sPDGFRβ, AD biomarkers, and POD. Furthermore, multivariate binary logistic regression was conducted as a sensitivity analysis to explore result stability and construct correction models, encompassing variables such as sex, age, years of education, MMSE scores, and additional factors, including the history of diabetes, hypertension, drinking, and smoking.

For better comprehension of the link between AD biomarkers in CSF sPDGFRβ and POD, mediation effect analyses were undertaken. Three mediation models were established: (1) CSF sPDGFRβ→CSF Aβ42→POD (CSF Aβ42 mediates brain pericyte injury); (2) CSF sPDGFRβ→CSF Ptau→POD (CSF Ptau mediates brain pericyte injury); (3) CSF sPDGFRβ→CSF Ttau→POD (CSF Ttau mediates brain pericyte injury). Mediation effects were deemed significant if *p* < 0.05. Causal mediation tests were executed using the “mediate” function within the “mediation” package in R.

As an exploratory analysis for clinical utility, this study generated receiver operating characteristic (ROC) curves and determined the area under the curve (AUC) to assess the predictive efficacy of CSF sPDGFRβ and AD biomarkers in anticipating POD.

## 3 Results

A total of 60 patients opted out of the scale test, 10 individuals had their surgeries canceled, and 23 participants could not provide CSF samples due to changes in anesthesia methods, puncture failures, and other reasons. Consequently, these cases were excluded, and the study eventually included 532 participants (Figure 1). All participants demonstrated normal cognitive function and underwent assessments for both CSF sPDGFRβ and CSF‐associated protein levels.

### 3.1 Participant Characteristics

**FIGURE 2:**
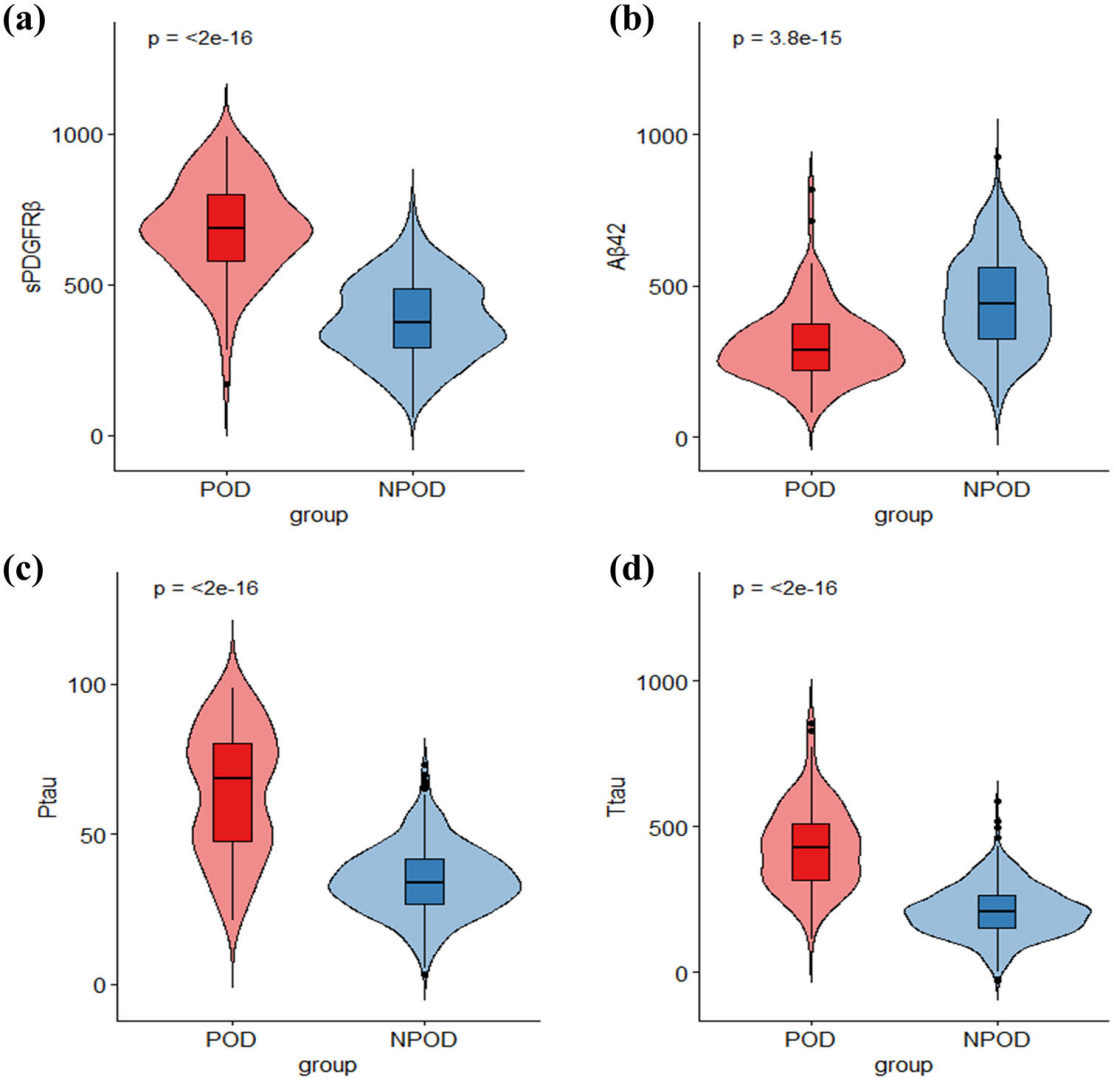
CSF sPDGFRβ and CSF AD biomarkers are related to POD. (a) Violin plot showing significantly higher levels of CSF sPDGFRβ in POD compared to NPOD (*p* < 0.05). (b) Violin plot showing significantly lower levels of CSF Aβ42 in POD compared to NPOD (*p* < 0.05). (c) Violin plot showing significantly higher levels of CSF Ptau in POD compared to NPOD (*p* < 0.05). (d) Violin plot showing significantly higher levels of CSF Ttau in POD compared to NPOD (*p* < 0.05).

Table 1 displays the characteristics of each participant. A total of 96 individuals experienced POD, resulting in an incidence rate of 18.05%, aligning with findings from prior research (Miller et al. 2018). Participants were categorized into either the POD group or the NPOD group based on the presence of POD. In comparison to the NPOD group, the POD group exhibited higher age, lower overall educational attainment and MMSE scores, elevated MDAS scores, and increased prevalence of diabetes and alcohol consumption (*p* < 0.05). Notably, there were significant variations in CSF sPDGFRβ, CSF Aβ42, CSF Ptau, and CSF Ttau (*p* < 0.05) (Table 2, Figure 2).

**TABLE 1:** Characteristics of participants.

Variable	POD (*n* = 96)	NPOD (*n* = 436)	*p*
Age [year, *M*(*Q*)]	66 (16.25)	59 (10)	0.000^*^
Male [*n* (%)]	68 (70.83)	271 (62.16)	0.109
Education [year, *M*(*Q*)]	7 (5)	16 (5)	0.000^*^
MMSE scores [*M*(*Q*)]	28 (1.75)	28 (3)	0.001^*^
Smoking [n(%)]	27 (28.13)	147 (33.72)	0.291
Drinking [n(%)]	31 (32.29)	62 (14.22)	0.000^*^
Hypertension [n(%)]	27 (28.13)	113 (25.92)	0.701
Diabetes [n(%)]	54 (56.25)	139 (31.88)	0.000^*^
MDAS scores [*M*(*Q*)]	11.50 (19)	1 (2)	0.000^*^

*Note*: Data are expressed as the Median (Interquartile spacing) [*M*(*Q*)] for numerical variables or as the count (%) for categorical variables unless special illustration.

Abbreviations: MDAS, the Memorial Delirium Assessment Scale.; MMSE, mini‐mental state examination; POD, postoperative delirium.

*p < 0.05.

**TABLE 2:** Comparison of sPDGFR and AD biomarkers between POD group and NPOD group.

Variable	POD (*n* = 96)	NPOD (*n* = 436)	*p*
sPDGFRβ [pg/mL, *M*(*Q*)]	684.86 (224.14)	375.20 (195.55)	0.000^*^
Aβ42 [pg/mL, *M*(*Q*)]	287.86 (155.62)	440.77 (237.65)	0.000^*^
Ptau [pg/mL, *M*(*Q*)]	68.27 (33.63)	33.90 (14.84)	0.000^*^
Ttau [pg/mL, *M*(*Q*)]	428.88 (203.09)	206.77 (113.57)	0.000^*^

*Note*: Data are expressed as the Median (Interquartile spacing) [*M*(*Q*)] for numerical variables.

Abbreviations: Aβ42, β‐amyloid42; POD, postoperative delirium; Ptau, phosphorylated tau.; sPDGFRβ, soluble platelet‐derived growth factor receptor β; Ttau, total tau.

*p < 0.05.

### 3.2 CSF sPDGFRβ and AD Biomarkers Are Associated With POD

In the logistic regression analysis, subsequent to adjusting for various confounding factors, heightened concentrations of CSF sPDGFRβ (odds ratio [OR] = 1.014, *p* < 0.05), CSF Ptau (OR = 1.136, *p* < 0.05), and CSF Ttau (OR = 1.018, *p* < 0.05) were identified as risk factors for POD. Conversely, elevated levels of CSF Aβ42 (OR = 0.994, *p* < 0.05) were observed to be a protective factor against POD (Table 3).

**TABLE 3:** Logistic regression on analysis and sensitivity analysis.

	Model 1^a^		Model 2^b^		Model 3^c^	
	OR (95%CI)	*p*	OR (95%CI)	*p*	OR (95%CI)	*p*
sPDGFRβ, pg/mL	1.015 (1.012–1.018)	0.000^*^	1.014 (1.010–1.017)	0.000^*^	1.014 (1.010–1.0018)	0.000^*^
Aβ42, pg/mL	0.993 (0.991–0.995)	0.000^*^	0.994 (0.991–0.997)	0.000^*^	0.994 (0.991–0.997)	0.000^*^
Ptau, pg/mL	1.116 (1.093–1.140)	0.000^*^	1.137 (1.095–1.181)	0.000^*^	1.136 (1.092–1.181)	0.000^*^
Ttau, pg/mL	1.018 (1.014–1.021)	0.000^*^	1.018 (1.013–1.023)	0.000^*^	1.018 (1.013–1.023)	0.000^*^

Abbreviations: 95%CI, 95% confidence interval; Aβ42, β‐amyloid42; OR, odds ratio; Ptau, phosphorylated tau; sPDGFRβ, soluble platelet‐derived growth factor receptor β; Ttau, total tau.

^a^ Model 1: Unadjusted.

^b^ Model 2: Adjusted for age, sex, education, and MMSE scores.

^c^ Model 3: Adjusted for age, sex, education, MMSE scores, smoking, drinking, hypertension, and diabetes.

*p < 0.05.

### 3.3 AD Biomarkers Mediate the Association Between CSF sPDGFRβ and POD

**FIGURE 3:**
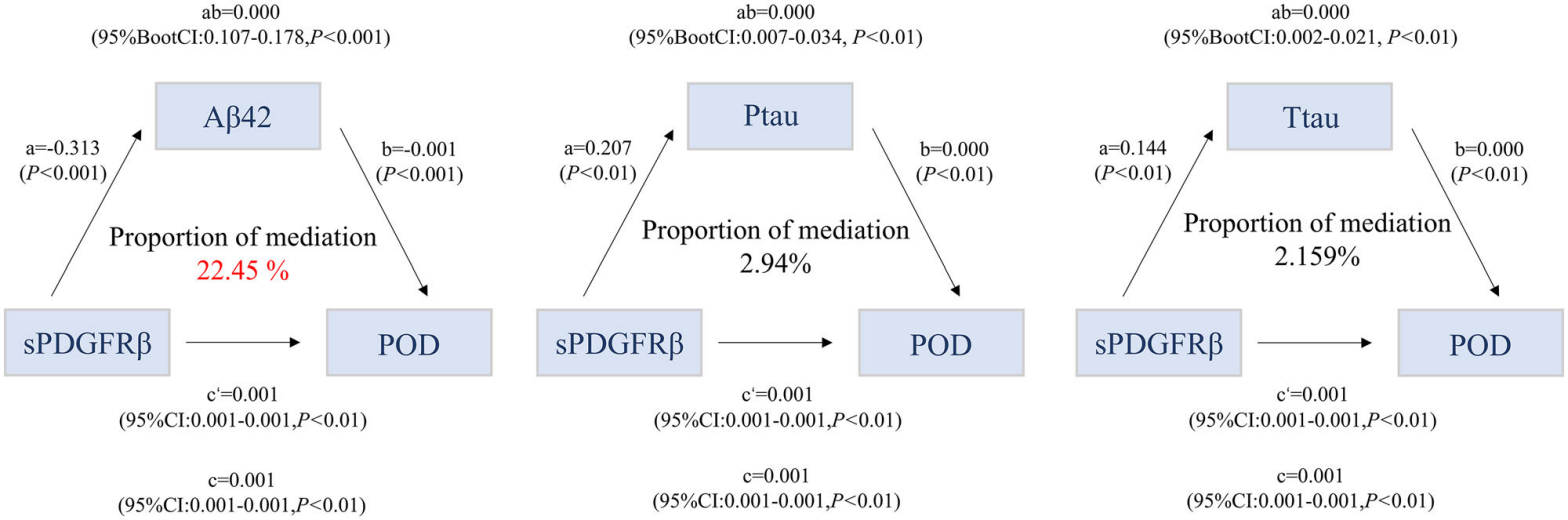
Analysis of the mediating effect of CSF AD biomarkers. Mediation analysis included the following variables: the levels of CSF AD biomarkers were treated as a mediator, POD was set as the dependent variable, and CSF sPDGFRβ was set as the independent variable. A value of *p* < 0.05 was considered statistically significant.

In examining the impact of CSF sPDGFRβ on AD biomarkers and POD, we established three mediation models. The results showed that CSF Aβ42 (22.45%) played a pivotal role as a mediator in the pathway from CSF sPDGFRβ to POD, while the mediating effects of CSF Ptau and Ttau were not significant. This implies that the observed effect may be predominantly influenced by Aβ protein involvement (Figure 3).

### 3.4 ROC Curve Predicts POD

**FIGURE 4:**
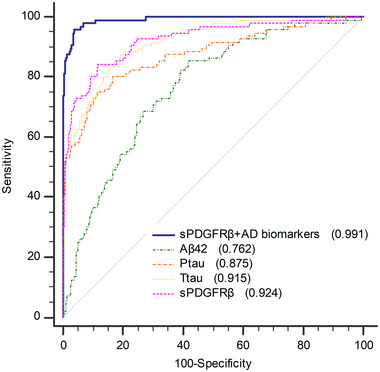
ROC curve. CSF sPDGFRβ + CSF AD biomarkers (AUC = 0.991); CSF sPDGFRβ (AUC = 0.924); CSF Ttau (AUC = 0.915); CSF Ptau (AUC = 0.875); CSF Aβ42 (AUC = 0.762).

The ROC curve demonstrated the efficacy of multiple factors in predicting POD. When compared to AD biomarkers or CSF sPDGFRβ in isolation, the combination of AD biomarkers and CSF sPDGFRβ proved to be more proficient in predicting POD (AUC = 0.991, *p* < 0.05). This finding holds potential as a reference for future clinical interventions (Figure 4).

## 4 Discussion

This study found an association between CSF sPDGFRβ and POD, suggesting it as a risk factor for the occurrence of POD. Notably, CSF Aβ42 was identified as a primary mediator in the influence of CSF sPDGFRβ on POD. Furthermore, we identified that the combined use of CSF sPDGFRβ and AD biomarkers was more effective in predicting POD.

POD, a common complication in surgical patients of old age, is linked to poorer functional recovery, extended hospital stays, and elevated short and long‐term mortality rates (Chen et al. 2022). CSF AD biomarkers (Aβ42, Ptau, and Ttau) contribute to cognitive dysfunction by influencing neurodegeneration (Mielke et al. 2014). Tau protein, generating Ptau through posttranslational modifications, plays a pivotal role in the formation of neurofibrillary tangles (Selkoe 2008). Anomalous hyperphosphorylation of tau disrupts microtubule stability, indispensable for synaptic plasticity and standard brain function (Busche and Hyman 2020; Wiberg et al. 2022). Aβ aggregation leads to amyloid plaques, triggering harmful reactions, neuronal degeneration, and death (Thal et al. 2015). The findings of this investigation revealed statistically significant distinctions in CSF Aβ42 and tau protein levels between the POD group and the NPOD group, aligning with outcomes from prior research (Wu et al. 2018; Huang et al. 2018).

The disruption of the BBB and the depletion of brain pericytes could potentially induce cognitive impairment. The BBB is a crucial selective semipermeable barricade sustaining the internal environment of the brain (Daneman and Prat 2015). Pericytes, positioned on capillaries, govern various microvascular functions and express PDGFRβ, pivotal for cerebrovascular stability (Cheng et al. 2018). Elevated CSF sPDGFRβ indicates damage at the pericellular level and the breakdown of the BBB, early indicators of cognitive dysfunction in humans (Sweeney et al. 2020). After adjusting for multiple confounding factors, this research displayed heightened CSF sPDGFRβ in individuals with POD, proposing BBB impairment due to brain pericyte injury as a contributing factor to POD.

For additional validation, we investigated the role of AD biomarkers in the correlation between CSF sPDGFRβ and POD. The results showed that the level of CSF sPDGFRβ in the POD group was increased, while the level of Aβ42 was decreased, suggesting a certain correlation between pericellular damage and Aβ deposition. Aβ40 and Aβ42 excretion through the BBB is recognized as a major clearance mechanism in the brain (Tarasoff‐Conway et al. 2015; Zlokovic 2005), suggesting that Aβ transport in the BBB may be impaired. The degeneration of pericytes and downregulation of LRP‐1 are fundamental mechanisms underlying BBB impairment in AD, influencing Aβ clearance (Ma et al. 2018). Absorption and clearance of CSF Aβ occurs through the circulatory and lymphatic systems. These processes depend on choroid plexus production of CSF, blood‐CSF barrier structural integrity, associated transporters, arachnoid villus resistance, and CSF lymphatic absorption. BBB dysfunction leads to the deposition of Aβ by increasing the production of Aβ and preventing its normal transport through BBB. Using transgenic mice that overexpress amyloid precursors, researchers have demonstrated that BBB integrity destruction precedes neuronal degeneration, and that early BBB damage is independent of Aβ and tau protein pathology (Saito et al. 2014). Under physiological conditions, complete BBB function can promote the clearance of Aβ and prevent the transfer of peripheral Aβ to the brain, avoiding the toxic accumulation of Aβ. Previous studies have shown that patients with mild cognitive impairment and AD have a large decrease in pericytes in the hippocampus, disruption of tight connections, and increased BBB permeability (Preis et al. 2024). Animal models of peri‐knockout cell markers‐sPDGFRβ showed significant disruption of BBB integrity, reduced density of neurovascular pairs and capillaries, reduced local cerebral blood flow, and significantly promoted pathological deposition and neuronal loss (Liu et al. 2023). More importantly, the increased Aβ neurotoxicity further destroys the structural basis, molecular regulation, and immune response of BBB, forming a vicious cycle and further aggravating the damage of BBB (Wang et al. 2021). In conclusion, there is an obvious interaction between BBB and POD pathology, and the increase in CSF sPDGFRβ is associated with an increased risk of POD due to BBB dysfunction and reduced Aβ42 clearance, and they promote each other to form a vicious cycle.

To the best of our knowledge, this is the first study to explore the link between CSF sPDGFRβ and POD, presenting a fresh benchmark for prognosticating POD. Nonetheless, it presents several limitations: (1) The sample size was comparatively small and derived from a single‐center clinical cohort, predominantly enrolled in eastern China. Achieving more precise and universally applicable results necessitates a more extensive population‐based multicenter clinical analysis. (2) Essential biomarkers for BBB leakage were not accessible. (3) This study was a cross‐sectional study that failed to follow up the POD population and lacked a relationship between pericellular damage and POD fluctuations.

## 5 Conclusion

In conclusion, this study examined the association between AD biomarkers, sPDGFRβ, and POD by exploring the changes of sPDGFRβ levels in CSF of POD cases and evaluated its predictive efficacy. These findings suggest that the increase in CSF sPDGFRβ is associated with an increased risk of POD due to BBB dysfunction and reduced Aβ42 clearance. Additionally, the combination of CSF sPDGFRβ with CSF AD biomarkers exhibits improved predictive abilities for POD, offering a valuable reference for future clinical prevention and intervention efforts.

## Author Contributions

**Zongfeng Guo**: conceptualization, data curation, investigation, writing – original draft. **Chen Zhang**: formal analysis, investigation, project administration, writing – original draft. **Xiang Wang**: formal analysis, validation, writing – original draft. **Weiguo Chen**: methodology, software. **Zongxiao Guo**: supervision, resources, writing – review and editing.

## Ethics Statement

Approval for the study was granted by the Ethics Committee of the Hai ‘an People’s Hospital Affiliated to Nantong University based on informed consent. This study complies with the Declaration of Helsinki.

## Consent

Informed consent was received from all participants.

## Conflicts of Interest

The authors declare no conflicts of interest.

### Peer Review

The peer review history for this article is available at https://publons.com/publon/10.1002/brb3.70436

## Data Availability

All data needed to evaluate the conclusions in the paper are present in the paper. Additional data that support the findings of this study are available from the corresponding author upon request.
